# Nasopharyngeal Foreign Body with Unusual Presentation

**DOI:** 10.22038/IJORL.2023.75783.3537

**Published:** 2024-01

**Authors:** Pratik Kumar, Meenakshi Sachdeva, Nandini Shruti

**Affiliations:** 1 *Department of Otorhinolaryngology, * *Maulana Azad Medical College, New Delhi, India.*; 2 *Department of Dermatology, Deep Chand Bandhu Hospital, Delhi, India. *

**Keywords:** Nasopharynx, Foreign body, Unusual, Rare

## Abstract

**Introduction:**

An unusual nasopharyngeal foreign body in a very young child with no clinical symptoms is a rare case presentation.

**Case Report:**

A nine-month-old child presented with a suspected history of foreign body ingestion without any clue to the parents about the nature of the foreign body. X-ray of the nasopharynx revealed a sharp unusual metallic “Louis Vuitton” shoe logo that the child had accidentally inserted into the nasopharynx via the oral cavity while playing. Foreign body was removed under general anesthesia without complications.

**Conclusion:**

X-ray nasopharynx should be included in the examination of a suspected case of foreign body ingestion, as an unusual shape of foreign body can even produce no clinical symptoms but still pose a potential life threat due to its dislodgement into the airway if missed or delayed.

## Introduction

Ingestion of foreign body is commonly seen in children. Depending on the size, shape and nature of the foreign body, it can lodge either in the nasal cavity, nasopharynx, oropharynx or even down into the esophagus. Children with nasal cavity foreign bodies are commonly seen as an otolaryngology emergency (1). 

Lodgment of an unusually sharp foreign body into the nasopharynx via the oral cavity is extremely rare. Patients with suspected nasopharyngeal foreign body can present with nasal obstruction, nasal discharge, snoring or nasal bleeding. Occasionally, patients can sometimes present with acute onset respiratory distress in cases of a large nasopharyngeal foreign body obstructing the choana and oropharynx.

## Case Report

A nine-month-old female child was referred to the otolaryngology emergency of our hospital with a suspected history of foreign body ingestion. History from their parents revealed that they had not seen the incident**,** nor were aware of it but were ambiguous due to the information from other children while playing. The child was sleeping comfortably with no symptoms on presentation. 

The general condition was fair, with pulse oximetry showing 100% oxygen saturation. On auscultation, air entry was bilaterally equal, as documented by the pediatrician. On clinical examination, the nasal cavity, oral cavity and oropharynx were clear. Diagnostic nasal endoscopy was done with a 2.7mm, 0-degree endoscope, which showed a sharp metallic foreign body lodged partially into the left choana in the nasopharynx. The foreign body was not completely visualized, and the type of foreign body was still doubtful. We referred the child for X-ray soft tissue neck lateral view and anteroposterior view to confirm the exact location and type of foreign body. 

**Fig 1:**
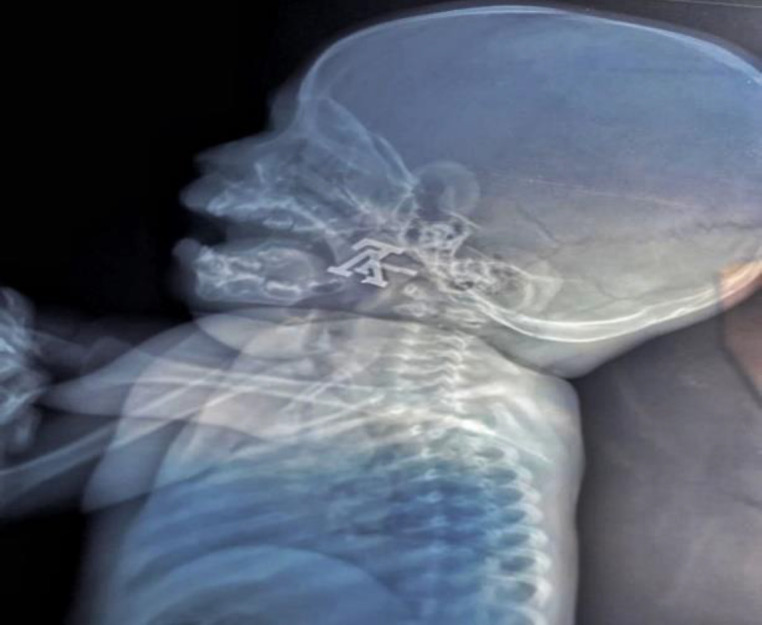
X-ray soft tissue neck (lateral view) showing radio-opaque foreign body in nasopharynx

To our surprise, this foreign body was a sharp metallic object that was lodged in the nasopharynx (Figure .1). 

**Fig 2:**
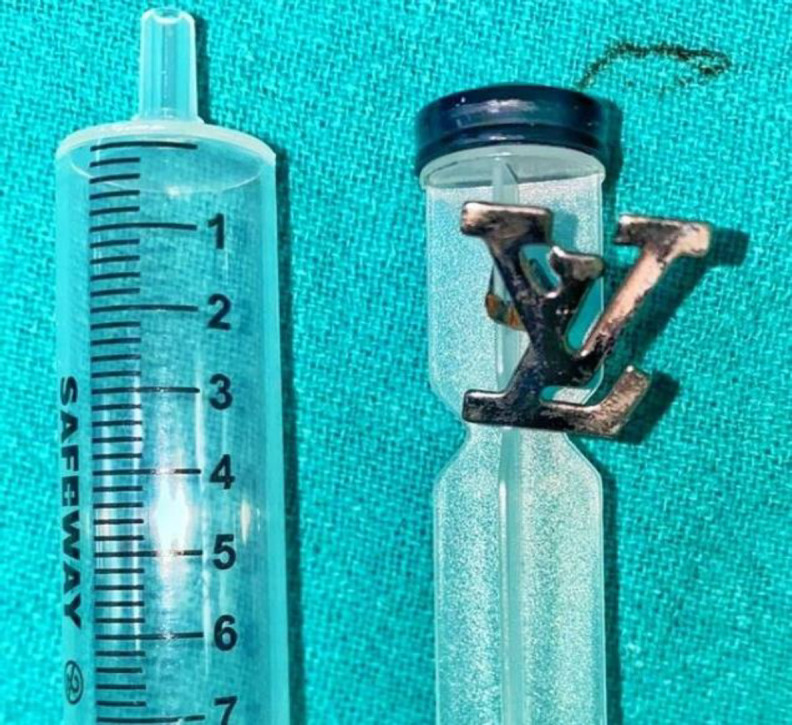
A metallic foreign body removed measuring 3 x 3 cm

We immediately planned the child for foreign body removal under general anesthesia. After sending all the basic blood parameters, the child was put on nil per oral and started on intravenous fluids. Nasal endoscopy and oral examination done under general anesthesia revealed a sharp foreign body with two flanges, one embedded into the left choana and the other into the soft palate posteriorly. During the procedure, an infant feeding tube was put into each nasal cavity and withdrawn from the oral cavity to temporarily retract the soft palate, dislodging one flange from the soft palate. Using 0^o ^nasal endoscope, a smallest- size eustachian tube catheter was inserted under vision into the left nasal cavity to dislodge the 2^nd^ flange embedding into the left choana. With the 70-degree angled endoscope, the foreign body was ensured to be free into the nasopharynx and a Negus artery forceps was used to grasp the sharp foreign body under vision and to remove gently via the oral cavity. The foreign body was a sharp metallic “Louis Vuitton” shoe logo that the child had accidentally inserted into the nasopharynx via the oral cavity while playing. (Figure.2).

Procedure was uneventful and the child was extubated successfully. Post-op x-ray soft tissue neck was clear. The child was kept under observation for 24 hours and was later discharged on oral medications.

## Discussion

Children with nasal cavity foreign bodies are one of the most common otolaryngology emergencies. These ingested or inhaled foreign bodies can lodge into the upper airway, esophagus or even bronchus. Children’s habit of putting everything into their mouth while playing as a form of developmental curiosity makes them more susceptible and prone to foreign body ingestion than adults. 

A foreign body in the nasopharynx is a rare, and our case had a very unusual and interesting foreign body stuck in a very young child. Another aspect that makes this an interesting case is the unusual symptom. Due to the unique shape of the foreign body, it was stuck into the nasopharynx without causing any signs of respiratory distress, nasal discharge, nasal blockage, or even pain. It’s unusual how an ingested foreign body could impact the nasopharynx instead of going into the trachea or esophagus. This can be explained by the possibility of a severe cough after foreign body ingestion, which can cause the foreign body to dislodge into the nasopharynx (2). The nasopharynx often serves as the hidden site for ingested foreign bodies and it has been suggested to include this in the clinical examination if swallowed foreign bodies are not found anywhere else (3). A literature review revealed a few unusual foreign bodies into the nasopharynx, like a large screw with its nut (4), gold ring embedded into the soft palate or a long-impacted marble into the nasopharynx (5,6), but all the cases had some symptoms on presentation. 

These unusual nasopharyngeal foreign bodies serve as the potential threat of dislodging and blocking the airway, leading to sudden airway compromise that can have some fatal consequences. Hence, if any child presents in an emergency with a suspected history of foreign body ingestion, routine X-rays of the nasopharynx should be included in the complete examination.

## Conclusion

Since an ingested or inhaled foreign body can dislodge into the nasopharynx, an X-ray of the nasopharynx should be included in the complete examination of a suspected foreign body in a young child. Unusual shapes of foreign bodies lodged in the nasopharynx can even present with no symptoms in a very young child. This way, we can avoid life-threatening potential consequences due to the possibility of dislodging a foreign body into the airway. 
